# The Maternal Cytokine and Chemokine Profile of Naturally Conceived Gestations Is Mainly Preserved during *In Vitro* Fertilization and Egg Donation Pregnancies

**DOI:** 10.1155/2015/128616

**Published:** 2015-08-09

**Authors:** Alicia Martínez-Varea, Begoña Pellicer, Vicente Serra, David Hervás-Marín, Alicia Martínez-Romero, José Bellver, Alfredo Perales-Marín, Antonio Pellicer

**Affiliations:** ^1^Department of Obstetrics and Gynecology, University & Polytechnic Hospital La Fe, 46026 Valencia, Spain; ^2^Department of Obstetrics and Gynecology, Valencia General University Hospital, 46014 Valencia, Spain; ^3^Valencian Infertility Institute, University of Valencia, 46015 Valencia, Spain; ^4^Department of Pediatrics, Obstetrics & Gynecology, Faculty of Medicine, University of Valencia, 46010 Valencia, Spain; ^5^Biostatistics Department, La Fe Health Research Institute, University & Polytechnic Hospital La Fe, 46026 Valencia, Spain; ^6^Cytomics Laboratory, Príncipe Felipe Research Centre, 46012 Valencia, Spain

## Abstract

This prospective longitudinal study aimed at comparing maternal immune response among naturally conceived (NC; *n* = 25), *in vitro* fertilization (IVF; *n* = 25), and egg donation (ED; *n* = 25) pregnancies. The main outcome measures were, firstly, to follow up plasma levels of interleukin (IL) 1beta, IL2, IL4, IL5, IL6, IL8, IL10, IL17, interferon gamma, tumor necrosis factor-alpha (TNF*α*), transforming growth factor-beta (TGF*β*), regulated upon activation normal T-cell expressed and secreted (RANTES), stromal cell-derived factor 1 alpha (SDF1*α*), and decidual granulocyte-macrophage colony-stimulating factor (GM-CSF) during the three trimesters of pregnancy during the three trimesters of pregnancy; secondly, to evaluate if the cytokine and chemokine pattern of ED pregnant women differs from that of those with autologous oocytes and, thirdly, to assess if women with preeclampsia show different cytokine and chemokine profile throughout pregnancy versus women with uneventful pregnancies. Pregnant women in the three study groups displayed similar cytokine and chemokine pattern throughout pregnancy. The levels of all quantified cytokines and chemokines, except RANTES, TNF*α*, IL8, TGF*β*, and SDF1*α*, rose in the second trimester compared with the first, and these higher values remained in the third trimester. ED pregnancies showed lower SDF1*α* levels in the third trimester compared with NC and IVF pregnancies. Patients who developed preeclampsia displayed higher SDF1*α* plasma levels in the third trimester.

## 1. Introduction

Maternal immunological tolerance is essential for successfully establishing and maintaining pregnancy [1, 2]. In early pregnancy decidua, type 1 inflammatory T-cells significantly decrease, whereas both type 2 cells and CD56^bright^ CD16^−^ cytokine-secreting uterine natural killer (uNK) cells significantly increase compared with the nonpregnant endometrium [3]. Thus, uNK cells (70%), macrophages (15%), and T-cells (10%) are the most abundant decidual leukocyte populations in early pregnancy [4–7]. Interestingly, a high CD4+CD25^bright^ T regulatory/T helper (Th) 17 cells ratio is also found in decidua in early human pregnancy [8]. Women with uncomplicated early pregnancies systemically display high serum Th2/Th1 cytokine [9] and CD4+CD25+Foxp3+ T regulatory (Treg)/Th17 cells ratios [10].

The maternal immune system has to tolerate the semiallogeneic fetus in naturally conceived (NC) and* in vitro* fertilization (IVF) pregnancies to allow favorable pregnancy outcome [1, 2]. In fact a shift toward peripheral Th1/Th2 augmentation [11] and Th17/Treg cells imbalance has been reported in women with recurrent pregnancy losses [10] and in pregnancy-specific diseases such as preeclampsia [2]. As the fetus is fully allogeneic to the mother in egg donation (ED) pregnancies, the maternal immune response should be more tolerogenic in these particular pregnancies [2, 12]. As a matter of fact, it has been hypothesized that defective maternal immune tolerance to the allogeneic fetus would lead to the development of pregnancy-specific diseases, which are more prevalent in ED, such as pregnancy-induced hypertension [1, 2].

Maternal immune response in ED pregnancies is different compared with NC gestations. Actually, ED and IVF pregnancies at term display higher levels of both CD4+CD25^bright^ regulatory T-cells and CD4+CD25^dim^ activated T-cells in maternal peripheral blood mononuclear cells compared with NC gestations. In particular, the ratio of T-activated : T regulatory cells in ED pregnancies is significantly lower than that in NC pregnancies. The percentage of T-activated cells in ED pregnancies correlates positively with the number of HLA mismatches. Interestingly, peripheral blood mononuclear cells in ED pregnancies do not show a higher alloreactivity to the allogeneic fetus. Regarding the maternal humoral immune response, pregnant women by ED at term display higher levels of IL10 and IL6 and lower levels of TGF*β* in serum [13]. Nevertheless, knowledge of the wide maternal humoral immune response against the allogeneic fetus in ED pregnancies is lacking. Thus the aim of this prospective study was to assess and contrast for the first time the maternal peripheral humoral immune response throughout pregnancy among NC, IVF, and ED pregnancies. Circulating maternal cytokines and chemokines of Th2 cells [interleukin (IL) 4, IL5, IL6, and IL10], Th1 cells [IL2, interferon-gamma (IFN*γ*), and tumor necrosis factor-alpha (TNF*α*)], Treg cells [IL10 and transforming growth factor-beta (TGF*β*)], Th17 cells (IL17), uNK cells [IL8, regulated upon activation normal T-cell expressed and secreted (RANTES), and stromal cell-derived factor 1 alpha (SDF1*α*)], proinflammatory cytokine IL1-beta (IL1*β*), and decidual granulocyte-macrophage colony-stimulating factor (GM-CSF) [2] were quantified. The maternal immune response of patients who developed preeclampsia was also evaluated and compared with the response of women with uneventful pregnancies. Finally, obstetric and perinatal outcomes were compared between pregnancies with a semiallogeneic fetus and those with fully allogeneic offspring.

## 2. Materials and Methods

### 2.1. Subjects

This prospective study was carried out in two University Centers in Valencia, Spain: University & Polythecnic Hospital La Fe and Valencian Infertility Institute. We studied 75 consecutive women whose pregnancy was achieved by three different modes of conception: NC (*n* = 25), IVF (*n* = 25), and ED (*n* = 25). Women were enrolled in the study at the time of their first pregnancy control in one of the aforementioned hospitals. Gestational age was based on the day of embryo transfer in ART pregnancies and on the last menstrual period in NC pregnancies, confirmed in all cases by a first trimester ultrasound scan. Exclusion criteria were multiple pregnancy, sperm donation, maternal autoimmune disease, and known maternal risk factors for preeclampsia: chronic arterial hypertension, chronic kidney disease, prepregnancy diabetes mellitus, thrombophilia, and history of preeclampsia in a previous gestation.

All women who achieved pregnancy by IVF or ED received vaginal progesterone, 400 mg/day, for the first 12 weeks of gestation. Patients were followed up at least quarterly until delivery, and pregnancy-related complications such as preeclampsia were gathered. The data of gestational age at delivery, weight of the newborn, and mode of delivery were also collected.

During pregnancy follow-up, 8 mL of peripheral blood was drawn from each patient between gestational weeks 11–14, 20–22, and 32–35 in one of the two hospitals. Two 4-mL EDTA tubes were stored at −20°C each time. Blood samples were centrifuged (3500 rpm for 10 min) at room temperature within the first 24 h after being taken, and the collected plasma was stored in 2-mL aliquots at −80°C.

This study was approved by the Ethics Review Boards of both participating hospitals. Written consent was obtained from each woman at the time of enrollment.

### 2.2. Cytokine and Chemokine Analysis

For cytokines detection in plasma samples, the MILLIPLEX MAP Technology (Merck Millipore, Germany) for Luminex 200 System (Luminex Corporation, USA) was used. The MILLIPLEX MAP Technology is a bead-based immunoassay capable of detecting up to 50 cytokines simultaneously in small sample volumes. The Luminex 200 System is a flow cytometry-based instrument equipped with two lasers, one for identifying cytokines and the other one for quantifying the concentration of the identified cytokine. The system uses Exponent 3.1 software to run and analyze samples (Luminex Corporation, USA).

Four different kits were used for the detection of 14 cytokines: Human Cytokine/Chemokine Magnetic Bead Panel (HCYTOMAG-60K) for RANTES, Human Cytokine/Chemokine Panel II (HCYP2MAG-62K) for SDF1*α*+*β*, TGF*β*1 Single Plex Magnetic Bead Kit (TGFBMAG-64K-01) for TGF*β*1, and Human High Sensitivity T-Cell Magnetic Bead Panel (HSTCMAG-28SK) for GM-CSF, IFN*γ*, IL10, IL17A, IL1*β*, IL2, IL4, IL5, IL6, IL8, and TNF*α*.

Samples were previously centrifuged at 1000 g for 1 min and the supernatant was collected to discard debris and excess lipids, which could interfere with the assay. For the Human Cytokine/Chemokine Panel II and Human High Sensitivity T-Cell kits, neat plasma samples were used. For the Human Cytokine/Chemokine kit, a 100-fold sample dilution was required. For the TGF*β*1 detection kit, samples had to be acidified and diluted (final dilution of 1 : 30) according to the kit instructions. Briefly, 25 *μ*L of samples (neat or pretreated) was incubated with antibody-immobilized beads in 96-wells plates overnight at 4°C. To perform the fluorescence labeling of the proteins bound to the beads, plates were washed and incubated with the detection antibodies and subsequently with the streptavidin-phycoerythrin reagent. Standard and control samples were used in each kit to develop the standard curve from which the quantitative analysis of samples was performed. Finally, plates were run in the Luminex 200 System following the protocol settings of the kits using Exponent 3.1 software. A sample analysis was performed with Exponent 3.1 software using the standard curves obtained in the acquisition for each kit. For this analysis, the fluorescence data measured during the samples acquisition were interpolated to the standard curve, and a quantitative result in protein concentration (pg/mL) was obtained.

### 2.3. Statistical Analysis

Numerical continuous data were expressed as the mean (standard deviation) and median (1st–3rd quartiles). Categorical data were shown as absolute and relative frequencies. A logarithmic transformation was applied to the concentration values of the different cytokines before any calculation. An exploratory heat map was built to view the evolution of the different cytokine concentration values throughout pregnancy in each group. To analyze our results, the inflammation status was summarized by performing two-group (high and low inflammation status) fuzzy clustering [14] on the cytokines data and using the computed GoM (grade of membership) to the high inflammation group as the summary variable. To better understand how the different cytokines related to the overall GoM, another heat map was built with the cytokine values, and the individuals were ordered according to their GoM score. Finally, a beta regression mixed model [15] was used to assess the association among inflammatory status and age, trimester, group, and other risk factors (such as body mass index (BMI) and nulliparity). Other bivariate analyses between groups were performed using Chi-square test for the categorical variables and *t*-test for continuous data. All the statistical analyses were performed using R (version 3.1.1) and the glmmADMB (version 0.8.0) [16] and cluster (version 1.15.2) R-packages.

## 3. Results 

### 3.1. Baseline Data

Women's baseline characteristics are presented in Table 1. Mean age was higher in the ED group, compared with NC and IVF pregnancies. No noticeable differences were found among groups for BMI upon the first pregnancy control, nulliparity, and number of previous pregnancies and miscarriages with the same partner.

The studied women did not have medical diseases and did not report receiving any medication upon the first pregnancy control except for one woman in the NC group and one in the IVF group who occasionally took benzodiazepines for anxiety.

### 3.2. Maternal Immune Response

**Figure 1:**
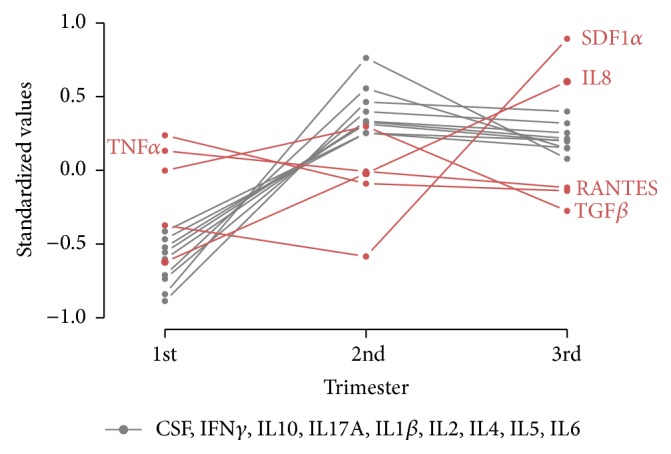
Evolution of the levels of each cytokine throughout pregnancy in the three study groups. Cytokine concentrations were expressed as standardized values (mean = 0, SD = 1). RANTES, regulated upon activation normal T-cell expressed and secreted; TNF*α*, tumor necrosis factor-alpha; TGF*β*, transforming growth factor-beta; IL8, interleukin 8; and SDF1*α*, stromal cell-derived factor 1 alpha.

**Figure 2:**
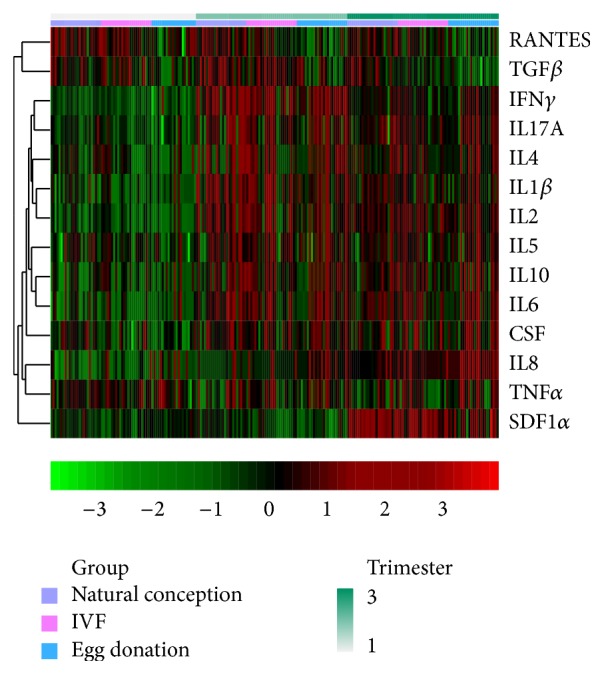
Heat map representing the cytokine and chemokine concentration values for each woman in all three groups throughout pregnancy. Whereas the three trimesters of pregnancy are represented on the first upper horizontal line, the three study groups in all three trimesters of pregnancy are represented on the second horizontal line. It can be observed that most cytokines and chemokines follow a similar pattern during pregnancy; their concentration rises in the second trimester and remains high in the third trimester of pregnancy. RANTES, regulated upon activation normal T-cell expressed and secreted; TGF*β*, transforming growth factor-beta; IFN*γ*, interferon-gamma; IL, interleukin 17, IL4, IL1*β*, IL2, IL5, IL10, and IL6; GM-CSF, decidual granulocyte-macrophage colony-stimulating factor; IL8; TNF*α*, tumor necrosis factor-alpha; and SDF1*α*, stromal cell-derived factor 1 alpha.

In NC pregnant women, there was a clear trend that the values of all the quantified cytokines, except RANTES, TNF*α*, IL8, TGF*β*, and SDF1*α*, rose in the second trimester, and these high values remained in the third trimester. IVF and ED pregnant women displayed a similar cytokine pattern throughout pregnancy (Figure 1). This can be seen in the exploratory heat map, which depicts the evolution of the cytokines measured in each woman throughout pregnancy (Figure 2). RANTES, TNF*α*, and TGF*β* showed similar values throughout pregnancy. In contrast, SDF1*α* and IL8 were the only cytokines whose values increased in the third trimester.

Subtle differences were found in the cytokine patterns for the three study groups. IL8 levels were higher in the three trimesters in ED and IVF pregnancies (*P* = 0.017 and *P* = 0.008, resp.) compared with NC pregnancies. In contrast, RANTES levels were lower in the three trimesters in ED and IVF pregnancies (*P* = 0.02 and *P* = 0.035) compared with NC gestations. Finally, SDF1*α* levels in the third trimester of ED pregnancies were significantly lower* versus* pregnancies with autologous oocytes (*P* = 0.004) (Table 2).

**Figure 3:**
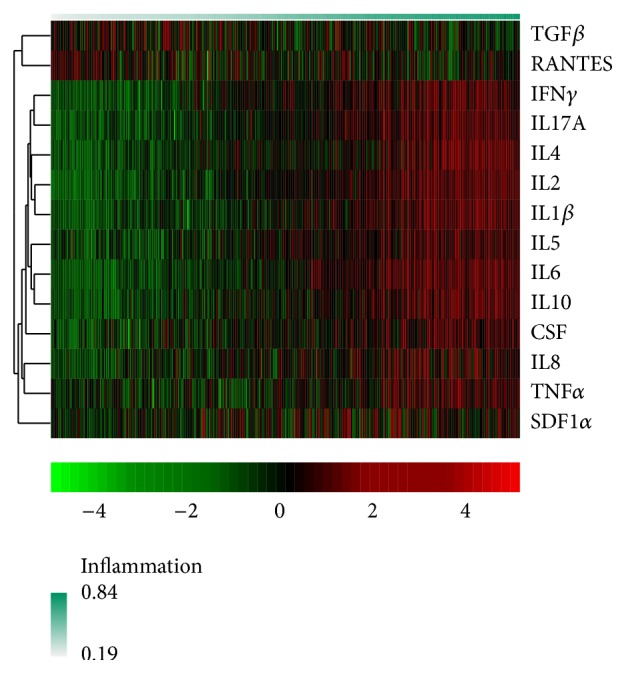
Heat map with the fuzzy clustering results. Individuals were ordered according to their GoM (grade of membership) score; a higher GoM score (red) denotes a higher inflammation status. TGF*β*, transforming growth factor-beta; RANTES, regulated upon activation normal T-cell expressed and secreted; IFN*γ*, interferon-gamma; IL, interleukin 17, IL4, IL1*β*, IL2, IL5, IL6, and IL10, GM-CSF, decidual granulocyte-macrophage colony-stimulating factor; IL8; TNF*α*, tumor necrosis factor-alpha; and SDF1*α*, stromal cell-derived factor 1 alpha.

**Figure 4:**
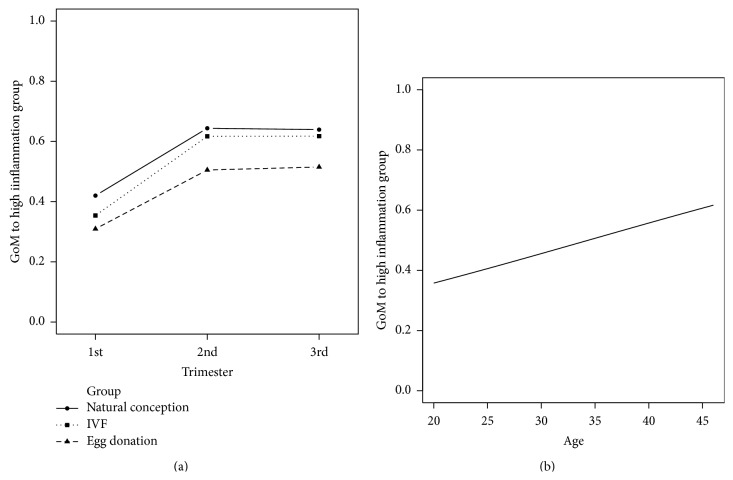
Effect plots for our beta regression mixed model results. (a) The three groups showed the same trend: their GoM (grade of membership) values increased in the second trimester and remained high in the third trimester. (b) A positive association between maternal age and GoM values.

The results of the fuzzy clustering performed to summarize inflammatory status are depicted in Figure 3. The women in the three study groups with higher IL1*β*, IL2, IL4, IL5, IL6, IL8, IL10, IL17A, TNF*α*, IFN*γ*, GM-CSF, and SDF1*α* values and lower RANTES and TGF*β* values were assigned higher GoM values in accordance with higher inflammatory status. The beta regression mixed model confirmed our initial findings in the exploratory heat map and showed a statistically significant association between the higher inflammatory statuses in the second and third trimesters (*P* < 0.001). There was no evidence of a differential evolution of the cytokine values among the three study groups (*P* = 0.38). A statistically significant association was found between older maternal age and higher inflammation status (*P* = 0.002). The effects of our regression model are represented in Figure 4.

### 3.3. Obstetric and Perinatal Outcome

Pregnancy-related complications are shown in Table 3. Incidence of first trimester vaginal bleeding was significantly higher in ED pregnancies. A nonsignificant trend of a higher incidence of gestational diabetes among ART pregnancies was noted particularly in older women (>40 years).

Given the small sample size, incidence of preeclampsia did not statistically differ among the three study groups (Table 3). Interestingly, the five women with preeclampsia displayed higher plasma SDF1*α* levels in the third trimester of pregnancy, compared with the average of their respective groups. No significantly different pattern was found for the remaining cytokines in preeclamptic patients compared with healthy pregnant women.

No differences were recorded among the groups for gestational age at delivery and birth weight. Only one fetus displayed intrauterine growth restriction whose mother was in the NC group and had preeclampsia. One ED pregnancy ended with a preterm birth, and the mother had preeclampsia and HELLP syndrome.

No congenital malformations were reported. Cesarean sections were more common among ART pregnancies, particularly in the ED group (Table 3).

## 4. Discussion

### 4.1. Principal Findings

The principal findings of this study are illustrated as follows. (1) The maternal cytokine and chemokine profile is preserved in ED pregnancies compared with the gestations with a semiallogeneic fetus. (2) The small number of patients who developed preeclampsia displayed higher plasma SDF1*α* levels in the third trimester of pregnancy compared with the healthy pregnant women of their respective study groups.

### 4.2. Cytokine and Chemokine Profile of NC, IVF, and ED Pregnancies

The maternal humoral immune response throughout pregnancy was similar in pregnancies with a semiallogeneic fetus (NC and IVF) compared with those with a fully allogeneic fetus (ED pregnancies). All the quantified cytokines, except RANTES, TNF*α*, IL8, TGF*β*, and SDF1*α*, increased in the second trimester and remained high in the third trimester.

RANTES levels were similar throughout pregnancy but showed lower values in the three trimesters in ED and IVF pregnancies compared with NC pregnancies. RANTES is the chemokine most synthesized by uNK cells at the human maternal-fetal interface. uNK cells play a key role in placental development as they regulate trophoblast invasion and vascular growth [7]. RANTES is also produced by the human endometrium during the implantation period [17–19] and by extravillous trophoblast cells (EVT) at 12–14 weeks of gestational age [20]. Considered to be one of the main chemokines to recruit T-cells [19, 21], it remains to be determined whether the lower RANTES concentrations during pregnancy in ED and IVF gestations, compared with NC ones, entail clinical implications.

Like RANTES, the TNF*α* levels did not increase in the second and third trimesters of pregnancy. Macrophages, lymphocytes, and trophoblast cells are the main producers of the proinflammatory cytokine TNF*α*, which also contributes to insulin resistance [22]. Preeclamptic patients display high circulating levels [23, 24] and placental expression of TNF*α* compared with women with uneventful pregnancies [25].

The IL8 levels constantly increased from the beginning to the end of pregnancy. In addition, ED and IVF pregnancies showed significantly higher IL8 levels throughout gestation. uNK cells are potent secretors of IL8 [7], which is a major neutrophil chemoattractant. Found in endothelial cells, it regulates endothelial cell proliferation, angiogenesis, and tumor growth. A role of IL8 in graft rejection has also been described [26, 27]. It is known that pregnancies complicated with preeclampsia display higher IL8 levels compared with uncomplicated gestations [23, 28–30]. The progressive rise in IL8 throughout pregnancy in all three groups may reveal its key role in cell proliferation and angiogenesis, both of which are required to successfully maintain pregnancy.

SDF1*α* levels increased in the third trimester compared with the first and second trimesters. Oddly enough, ED pregnant women presented significantly lower SDF1*α* levels in the third trimester than in the two other study groups. It is known that SDF1*α* is secreted by first trimester human trophoblast cells. It induces trophoblast proliferation, mediates crosstalk between trophoblasts and decidual stromal cells, and recruits uNK cells into the decidua through its interaction with chemokine (C-X-C motif) receptor 4 (CXCR4) [31]. Paradoxically, microarray analyses have revealed that it is the chemokine least synthesized by uNK cells in the first trimester of pregnancy [7]. A mice model has shown that SDF1*α* causes migration of Treg cells into the pregnant uterus [32]. Actually, SDF1*α* is considered as alloimmune biomarker [33], and its expression has been reported to be high in chronic human renal allograft rejection [34]. Thus the lower SDF1*α* levels in the third trimester of ED pregnancies, compared to gestations with autologous oocytes, may contribute to maintaining these pregnancies with a fully allogeneic fetus. Further studies are needed to clarify the role of SDF1*α* during gestation, particularly in ED pregnancies.

Finally, TGF*β* levels lowered in the third trimester of pregnancy compared with previous trimesters. TGF*β* is produced largely by uNK cells and plays a key role in angiogenesis and immunoregulation [35]. The anti-inflammatory cytokine TGF*β* is also produced by Treg cells [36]. The higher TGF*β* levels during the first and second trimesters may reflect the key role that uNK and Treg cells play in both the establishment and initial maintenance of pregnancy and the promotion of angiogenic and anti-inflammatory effects during this gestational period.

One unexpected finding in the three study groups was the high levels of most of the studied pro- and anti-inflammatory cytokines in the second half of pregnancy. The maternal immune response needs to be more intense at onset of pregnancy for proper fetal immune recognition and initial tolerance of pregnancy. However, this initial maternal immune response may take place locally in the basal decidua, which may not be associated with a systemic immune response in maternal blood. Even if this assumption was correct, it poses the question of why maternal plasma cytokines increase in the second half of pregnancy in uneventful gestations and which role this may play if there is any.

It has been suggested that pregnancies with a completely allogeneic fetus require an enhanced maternal immune tolerance in order to achieve a successful pregnancy [2, 12]. Actually, the percentage of FoxP3+ regulatory T-cells within the CD4+CD25^bright^ T-cells in peripheral blood of term pregnant women is higher in ED pregnancies compared with NC pregnancies [13]. The similar maternal Cytokine and Chemokine profile throughout pregnancies with allogeneic and semiallogeneic fetus further supports that regulatory T-cells (FoxP3+, CTLA-4+, and HLA-DR+) are crucial in maintaining human pregnancy.

### 4.3. Pregnancy Outcomes

Regarding pregnancy-related disorders, the incidence of first trimester vaginal bleeding was significantly higher in ED pregnancies compared with NC and IVF gestations. This is consistent with knowledge about the higher incidence of bleeding complications in the first trimester as a result of the more frequent placental pathology in ED pregnancies [12].

Compared with healthy pregnant women, it is known that preeclamptic women present similar plasma RANTES levels [29] but higher circulating levels of IL6, IL8, TNF*α*, IL2/IL4, IFN*γ*/IL4, and Th17/Treg cells [2]. In our series, the five women with preeclampsia had similar RANTES levels but raised levels of alloimmune biomarker SDF1*α* in the third trimester of pregnancy. The role of SDF1*α* in preeclampsia is still unknown. Given our small sample size, a higher incidence of preeclampsia was not found in ED pregnancies. Nevertheless, it is widely accepted that ED pregnancies are associated with a higher incidence of pregnancy-induced hypertension [12]. Thus further studies are required to elucidate if an abnormal maternal immune response against the allogeneic fetus in ED pregnancies contributes to the elevated incidence of preeclampsia in ED.

In spite of the small simple size, the ED group did not have worse pregnancy outcomes. This supports previous knowledge that incidence of perinatal complications in ED pregnancies is comparable to conventional IVF pregnancies [12].

### 4.4. Strengths and Limitations

The strength of the present study is the fact that, to the best of our knowledge, this is the first study to compare the maternal plasma cytokine profile among NC, IVF, and ED pregnancies. The limited sample size of the three study groups can be considered as study's main weakness. Moreover, although the maternal peripheral immune response throughout pregnancy provides prospective information about the tolerogenic response in both pregnancies with a semiallogeneic fetus and with an allogeneic fetus, further studies of the placenta are required to unravel the local maternal-fetal immune response particularly in ED pregnancies.

## 5. Conclusions

The maternal plasmatic cytokine profile of naturally conceived pregnancies was greatly conserved in IVF and ED pregnancies. Despite the limited sample size, the SDF1*α* levels were significantly lower in the third trimester of ED pregnant women. In addition, preeclamptic women displayed higher SDF1*α* levels in the third trimester. Further studies are required to confirm these findings and to assess the role of SDF1*α* in pregnancy and in pregnancy-related disorders such as preeclampsia.

## Figures and Tables

**Table 1:** Baseline characteristics of the women studied. Data are expressed as *N* (%) or mean (SD).

	Natural conception group	*In vitro* fertilization group	Egg donation group
Maternal age (years)	31.5 (4.7)	34.6 (3.3)	40.2 (2.7)
Body mass index (kg/m^2^)	24.4 (4.1)	23.3 (2.6)	25.2 (3.8)
Nulliparity	13 (52%)	24 (96%)	19 (76%)
Previous pregnancy with the same partner	13 (52%)	1 (4%)	3 (12%)
Previous miscarriage with the same partner	4 (16%)	6 (24%)	9 (36%)

**Table 2:** Values of IL8, RANTES, and SDF1*α* of the three study groups during pregnancy [mean (SD)].

Chemokine	Conception method	1st trimester	2nd trimester	3rd trimester
IL8	NC	0.18 (0.34)	0.29 (0.70)	0.61 (1.13)
	IVF	0.45 (1.18)	0.34 (0.59)	0.57 (0.54)
	ED	0.40 (0.68)	1.00 (1.77)	1.22 (1.29)

RANTES	NC	59005.1 (41368.2)	48423.2 (28486.7)	43258.1 (30534.9)
	IVF	47438.1 (31725.0)	30195.4 (19065.6)	27216.9 (16902.2)
	ED	35255.8 (27131.5)	18625.9 (17928.7)	21275.1 (17626.3)

SDF1*α*	NC	1424.0 (492.3)	1449.5 (377.9)	3329.7 (1007.8)
	IVF	1304.4 (511.5)	1194.0 (441.6)	3057.4 (1027.5)
	ED	1480.7 (375.2)	1089.8 (502.9)	2577.8 (2299.2)

NC: natural conception; IVF: *in vitro* fertilization; ED: egg donation pregnancy.

**Table 3:** Comparison of pregnancy outcome of the three study groups. Data expressed as *N* (%) or mean (SD).

	Natural conception group	*In vitro* fertilization group	Egg donation group	*P*
First trimester vaginal bleeding	4 (16%)	6 (24%)	15 (60%)	0.001
Preeclampsia	1 (4%)	2 (8%)	2 (8%)	0.81
HELLP syndrome	0 (0%)	1 (4%)	1 (4%)	0.60
Gestational diabetes	0 (0%)	3 (12%)	4 (16%)	0.075
Gestational age at delivery (weeks)	39.7 (**1.0**)	39.7 (**1.2**)	39.0 (**1.5**)	0.14
Preterm births	0 (0%)	0 (0%)	2 (8%)	0.13
Vaginal delivery	23 (92%)	12 (48%)	6 (24%)	0.004
Cesarean section	2 (8%)	13 (52%)	19 (76%)	0.001
Birth weight (g)	3286.8 (**536.3**)	3401.4 (**420.5**)	3282.3 (**504.1**)	0.62
Low birth weight (<2500 g)	2 (8%)	1 (4%)	1 (4%)	0.94
